# The relationship between plasma ferritin levels and body mass index among adolescents

**DOI:** 10.1038/s41598-018-33534-4

**Published:** 2018-10-17

**Authors:** Khulood K. Shattnawi, Mahmoud A. Alomari, Nihaya Al-Sheyab, Ayman Bani Salameh

**Affiliations:** 1 Jordan University of Science and Technology, Maternal & Child Health Nursing Department, Irbid, 22110 Jordan; 2 Jordan University of Science and Technology, Division of Physical Therapy, Department of Rehabilitation Sciences, Irbid, 22110 Jordan; 3 Qatar University, Division of Physical Education, Department of Educational Sciences, Doha, Qatar; 4 Al-Zaytoonah University of Jordan, Faculty of Nursing, Amman, 11733 Jordan

## Abstract

Circulatory Ferritin concentration varies with age, sex, and body composition. Studies that determine the relationship of different body weight measurements with plasma ferritin concentration in adolescents are lacking. A descriptive cross-sectional design was utilized. Data collection involved self-reporting demographics, blood samples, and body composition measures for a sample of 814 healthy Jordanian adolescents. Ferritin deficiency was observed in 55.8% of the study population. Simple linear regression showed that BMI, gender, location, and smoking status 2.5%, 3.9%, 0.4%, and 0.4%, respectively, associated positively with plasma ferritin level (p < 0.05). After controlling for gender, location, and smoking status, additional hierarchal multiple linear regression showed that BMI explained 2.2% of plasma ferritin (p < 0.000). However, the obesity-stratified hierarchal multiple linear regression, showed that BMI explained 2.1% of plasma ferritin in the overweight and obese (HI) adolescents (p = 0.02), but not in the under and normal weight (LO) adolescents (p = 0.91). After controlling for gender, location, and smoking status, the ANCOVA showed that plasma ferritin level was greater (p < 0.000) in the HI (19.00 ± 13.6) versus the LO (15.20 ± 10.4) obesity group. Our results indicated that normal ferritin level among obese people does not necessarily indicate normal iron storage.

## Introduction

Ferritin is a protein pivotal for various vital body organs, processes, functions, and diseases. It has been implicated in coronary artery disease and malignancy and directly involved in sideroblastic anemias, neurodegenerative disorders, inflammation, and hemophagocytic syndrome^1,2^. However, it is particularly essential for iron storing^3^ and supply^2^. Ferritin level ranges are 18–115 μg/L for women and 30–300 μg/L for men while the range is 7–140 μg/L in children^4^.

Obesity is spreading across the globe, primarily in developing countries^5^, including Jordan^6^. A recent report shows that adolescent overweight and obesity prevalence in Jordan is reaching 30%^7^, which is greater than those in developed countries^8^ and around the world^9^. Additionally, it has been linked to coronary artery disease, hypertension, hyperlipidemia, diabetes mellitus, and musculoskeletal, immune, and nervous system disorders, with a profound impact on quality of life^10^. In fact, obesity has recently been classified as a disease^5^.

Several studies, with contradictory findings, have detected an association between ferritin and obesity with few reports concluding that high ferritin levels were associated with increased obesity^11–13^. However, others reported either similar^14,15^ or even lower^16^ ferritin concentration in obese versus normal weight adults.

In adolescents, the relationship of ferritin with obesity is still elusive with only few studies showing an inverse association between serum ferritin level and obesity^2,16,17^. Therefore, the present study aimed at assessing the association of body mass index (BMI) with plasma ferritin concentration in a sample of apparently healthy 7^th^–10^th^ grade Jordanian students. Given previous results, we hypothesized that BMI is related to circulatory ferritin levels in adolescents. The results can help recognizing the importance of body composition for plasma ferritin levels.

## Results

### Participants

A total of 2691 adolescents gave parental consent and self-assent to participate in the study. Plasma ferritin level was obtained from 1046 while BMI was obtained from 2488 adolescents. As in Table 1, plasma ferritin and BMI measurements obtained concurrently from 873 adolescents. About half of the participants were females while the largest portions of the students were in the 9^th^ grade.

**Table 1:** The participant demographic (n = 873).

Gender (% male)	54.9
Age (yrs, mean ± SD)	14.6 ± 1.0
Weight (kg, mean ± SD)	56.3 ± 13.0
Height (cm, mean ± SD)	161.0 ± 9.0
Smoking status (%Yes)	65.3
Grade	
7 (%)	21.4
8 (%)	23.3
9 (%)	32.6
10 (%)	22.7
Location	
Rural (%)	52.6
Urban (%)	47.4
Family income (%)	
Above poverty	34.5
Below poverty	65.5
Mother education level (%)	
Above bachelor degree	37.9
Below associate degree	62.1
Father education level (%)	
Above bachelor degree	44.9
Below associate degree	55.1
Ferritin status (μg/L, mean ± SD)	16.1 ± 12.1
Below normal (%)	55.8
Normal (%)	44.2
Obesity status (BMI, mean ± SD)	21.6 ± 3.9
Normal and under weight	72.7
Over weight and obese	27.3

### Normality test

The normality test revealed that ferritin was not normally distributed (W-S p-value < 0.000). The values were then log-transformed and used for analysis.

### Relationship of Obesity Measures with Ferritin

The analysis revealed that 628 (72.7%) adolescents were in the LO while 236 (27.3%) adolescents were the HI obesity groups. Additionally, 382 (44.2%) adolescents were ferritin normal while 483 (55.8%) adolescents were ferritin deficient.

**Figure 1:**
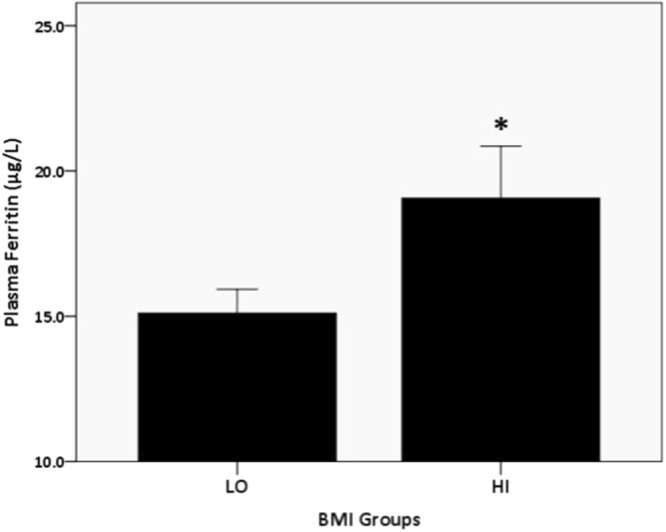
Relationship of BMI with plasma ferritin in the HI group. R^2^-change = 0.021; *p* < 0.02.

Individual simple linear regression for the entire sample showed that BMI, gender, location, and smoking status explained (*p* < 0.05) 2.5%, 3.9%, 0.4%, and 0.4%, respectively, of plasma ferritin level. However, father and mother education level, income, were not related (*p* < 0.05) to plasma ferritin level among the adolescent participants. Additional hierarchal multiple linear regression, after controlling for gender, location, and smoking status, showed that BMI explained 2.2% of plasma ferritin (*p* < 0.0001). However, as in Fig. 1, the obesity-stratified hierarchal multiple linear regression, showed that BMI explained 2.1% of plasma ferritin in the HI (*p* = 0.02), but not in the LO (*p* = 0.91), obesity groups.

**Figure 2:**
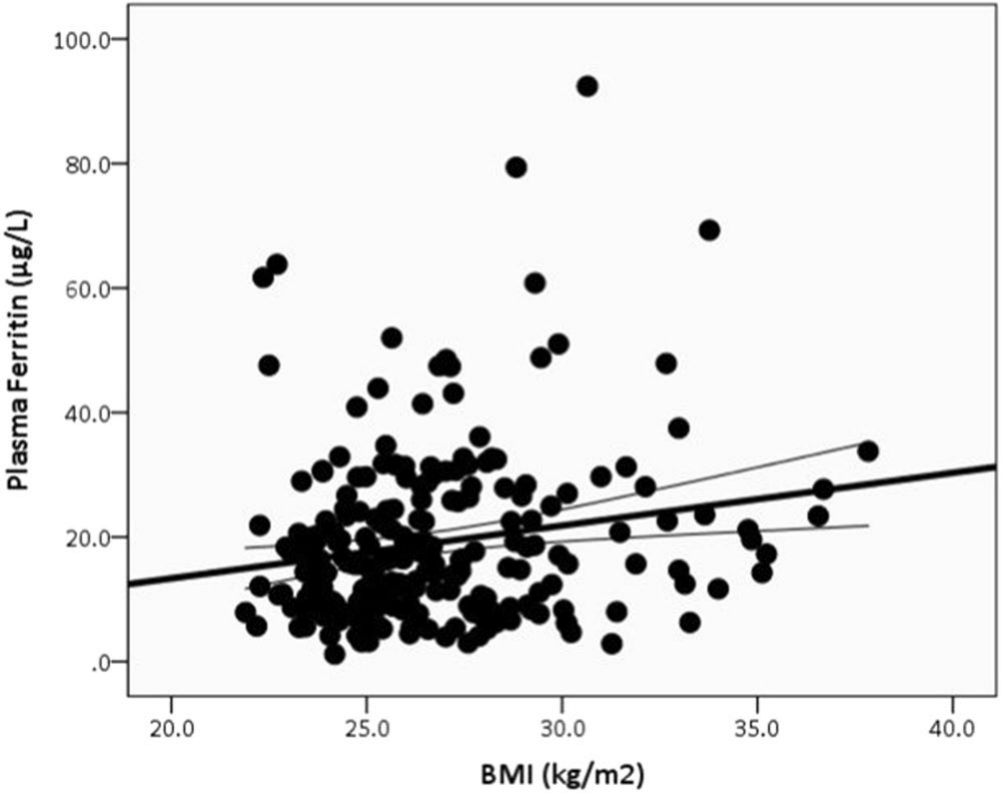
ANCOVA comparison in serum ferritin (μg/L) levels among high school students, after controlling for gender, smoking status, and location in the LO versus HI obesity groups. *The data are presented in mean* ± *SE*. *Indicate differences at p < *0.05*.

As in Fig. 2, the ANCOVA, after controlling for gender, location, and smoking status, showed that plasma ferritin level was greater (*p* < 0.000) in the HI (19.00 ± 13.6) versus the LO (15.20 ± 10.4) obesity group.

## Discussion

The study examined the relationship of plasma ferritin level with obesity among adolescents. Based on WHO classifications^4^, our results showed that more than half of the adolescents were ferritin deficient. Additionally, close to 1/3^rd^ of the adolescents were classified as either obese or overweight. Interestingly, the comparisons showed that plasma ferritin was greater in the obese and overweight versus the normal weight adolescents. Most importantly, the results showed that plasma ferritin level was positively associated with BMI in the obese but not in the normal weight individuals.

Worldwide, iron depletion is the most common nutrient deficiency among adolescents^18^. The literature has identified athletic participation, menstruation, poor diet, and mismatch between ferritin demand and availability as possible risk factors for iron deficiency among adolescents^19^. Iron deficiency is also associated with decreased exercise capacity due to earlier onset of fatigue than healthy counterpart, which may result in stationary lifestyle, and subsequently possible weight gain^11^. Low iron level may also lead to anemia and its sequentially health problems including that might cause cognitive impairment, low immunity, and high risk pregnancies^20^.

Another cause of obesity among adolescents is the consumption of unbalanced and unhealthy meals, which consequently leads to further decline in iron stores^21^. Poor nutrition among children from lower socioeconomic status has also been implicated. These underprivileged children tend to consume low-cost meals, which is usually rich in fat and sugar and low in essential nutrients, such as iron^22^. These findings, along with our findings, indicate a need for developing strategies to improve eating habits to help restrain obesity in this population.

Importantly, BMI showed positive association with plasma ferritin concentration among obese adolescents, confirming previous studies^13,14^, showing greater circulatory ferritin level among obese adolescents^13,14^. Huang *et al*.^12^ reported that BMI is associated positively with plasma ferritin but negatively with serum iron in overweight/obese adolescents compared to the normal weight or underweight adolescents. Thus, according to Huang *et al*. results, circulatory ferritin does not reflect iron deficiency in overweight/obese adolescents. Furthermore, the current and previous findings suggest that different reference values of ferritin level should be considered when evaluating iron deficiency in overweight and obese adolescents. In addition, the need for different measures to assess iron level in obese adolescents is warranted. Conversely, other studies reported similar ferritin concentrations between obesity groups^14^. A negative relationship between ferritin level and BMI was also reported in Iranian girls^17^. These contradictory findings highlight the need for further studies to define the relationship between ferritin and obesity.

The mechanism for the positive relationship of obesity with ferritin is not fully understood. There is a growing evidence supports that obesity-related inflammatory process can increase ferritin level^12,23^ even with depleted iron stores^24,25^. Serum ferritin is an acute phase reactant protein, similar to CRP, which increases in response to inflammatory processes in obese people, including adipokines released by adipocytes^2,16^. Inflammatory cytokines are activated in response to obesity, and accordingly, hepcidin, an iron regulatory protein, is released as a defense mechanism resulting in decreased iron level and increased ferritin level^17^. This indicate that inflammation-induced obesity results in iron deficiency, though ferritin is elevated. Therefore, further research is needed to evaluate the inflammatory markers along with ferritin levels in adolescents, especially obese ones. Longitudinal and experimental studies that include monitoring ferritin level simultaneously with iron stores in obese versus normal weight adolescents are required to verify these relationships. A biomarker of inflammation should also be measured at the same time.

Our findings have important clinical implications. Given the rapidly increasing adolescence obesity (27.3% of our sample were obese), with the known link between obesity and iron deficiency (55.8% of our sample were ferritin deficient), guidelines for screening for obesity and iron deficiency should be promoted and implemented for this population. Additionally, routine monitoring of ferritin and iron levels are warranted among obese individuals. Special precautions are also advised when using ferritin level as an indicator of iron deficiency in obese adolescents, especially ferritin level in obese adolescents seems not reflect body iron storage^26^. Strategies to decrease iron deficiency and obesity in this high-risk population imperative to minimize the negative effects of iron deficiency and obesity. These strategies include promoting regular exercise and sufficient nourishment.

As with other studies, our study has some limitations. The first is the use of a cross-sectional design, which limits inferring causal relationships that may be obtained from a longitudinal design. A second limitation is with the relatively small sample size from a small country that may limit the generalizability of our findings. Another limitation is the use of BMI only as an indicator of obesity/overweight among adolescents. Including more detailed measures of body fat and fat distribution may strengthen our findings. In addition, this study did not measure biomarkers of inflammation, which if measured may verify any existing relationships. However, the current findings may serve as a platform for future longitudinal studies.

## Conclusions

The results confirmed the positive association of obesity with plasma ferritin, especially among obese adolescents. Recognition of these results suggests taking this association into consideration when assessing iron deficiency for obese/overweight adolescents. Our results also imply that, although ferritin concentration in plasma/serum is the most pertinent indicator in monitoring iron stores in healthy persons^27^, it is of less value in assessing anemia in obese persons. However, more studies are needed to further establish ferritin relationship with various obesity measures using larger samples and longitudinal design.

## Methods

### Design, sampling and ethical approval

This is a descriptive cross-sectional study. The current study involved self-reporting and biometric data collection tools. The study was designed to assess the association of body mass index with circulatory plasma ferritin concentration in randomly selected adolescents in Jordan after being stratified for gender and school type. The inclusion criteria include students in grade 7–10, attends school regularly in any of the randomly selected schools, free of chronic diseases, and able to understand and write in Arabic fluently.

Written informed parental consents as well as informed child assents, approved by the required institutional review boards were obtained from all participants. Schools were contacted first and pre-arrangements with school principals were made in order to ensure that important teaching processes and school classes would not be interrupted. The study was approved and carried out in accordance with the guidelines and regulations of both the University of Science and Technology (JUST) Institutional Review Board (IRB) committee and the Jordanian Ministry of Education Ethics committee.

Study measurements were collected under university professor supervision and with the help of a trained research team and volunteers to ensure consistent and unified measurements. Self-reporting demographics were obtained in classes while blood samples and body composition measures were collected in the school nursing units. Blood withdrawals were performed by experienced nurses and medical laboratory technicians. Additionally, schoolteachers were available to facilitate data collection. All efforts were observed and precautions were taken to avoid possible risks that might be associated with data collection, especially before, during, and after blood withdrawal.

### Blood samples

Venous blood samples were drawn from the arm and collected in an EDTA tube for ferritin analysis. Plasma ferritin concentrations were determined using Beckman Coulter Access Immunoassay Systems (Access Ferritin). The plasma ferritin cutoff value of 15 μg/L was used according to the WHO^4^ set for the study’s sample age range.

### Physiological measures

A digital weighing scale was used to determine weight (Wt), while a standard tape measure at the umbilical level and widest portion of the buttocks was used to determine waist (W) and hip (H) circumferences, respectively, and height (HT). BMI was calculated using standard procedures^28,29^. BMI z-scores were calculated relative to Centers for Disease Control and Prevention gender-specific, BMI-for-age reference data^30^.

### Statistical analyses

The SPSS package (version 21.0; Chicago, IL) was used to statistically analyze the data. Alpha was set a priori at p ≤ 0.05. Continuous tabulated data are presented in means ± SD while presented in means ± SE in figures. The adolescents were stratified according to obesity using BMI measure, to low (LO) and high (HI) obesity. The LO group included the adolescents who were classified as under and normal weight while the HI group included over weight and obese students, according to the CDC recommendations^30^.

Several individual linear regression models for the entire sample and sub-analysis (obesity-stratified) were used to establish the relationship of BMI, gender, age, smoking status (yes versus no), location (urban versus rural), family income (below versus above poverty line), and father and mother education level (below associate versus above bachelor degrees) with plasma ferritin. Additional obesity-stratified hierarchal multiple linear regression after controlling for gender, location, and smoking status related to plasma ferritin, was used to examine the relationship of obesity with plasma ferritin. One-way ANCOVA was used to compare plasma ferritin levels between the LO and HI obesity groups, after controlling for variables related to plasma ferritin.

Ferritin values were examined for normality using Welk-Shapiro (W-S) test. The values were then log-transformed and used for analysis.
