# Author Correction: The relationship between plasma ferritin levels and body mass index among adolescents

**DOI:** 10.1038/s41598-018-37077-6

**Published:** 2019-01-24

**Authors:** Khulood K. Shattnawi, Mahmoud A. Alomari, Nihaya Al-Sheyab, Ayman Bani Salameh

**Affiliations:** 1 Jordan University of Science and Technology, Maternal & Child Health Nursing Department, Irbid, 22110 Jordan; 2 Jordan University of Science and Technology, Division of Physical Therapy, Department of Rehabilitation Sciences, Irbid, 22110 Jordan; 3 Qatar University, Division of Physical Education, Department of Educational Sciences, Doha, Qatar; 4 Al-Zaytoonah University of Jordan, Faculty of Nursing, Amman, 11733 Jordan

Correction to: *Scientific Reports* 10.1038/s41598-018-33534-4, published online 17 October 2018

The original version of this Article contained errors. The author Mahmoud A. Alomari was incorrectly listed as Mahmoud Alomari. As a result, the Author Contributions statement now reads:

“K.K.Sh., M.A.A., N.A. & A.B. contributed to the acquisition, analysis and interpretation of data. All authors drafted the article and approved the final version to be published.”

In addition, there were errors in the affiliation list, where Affiliation 1 was duplicated as Affiliation 3 and an additional affiliation for Mahmoud A. Alomari, ‘Qatar University, Division of Physical Education, Department of Educational Sciences, Doha, Qatar’ was omitted.

These errors have been corrected in the PDF and HTML versions of this Article.

